# Effect of Diet Supplementation with Oat Hay and Whole Carrot on Rabbit Growth and Productive Efficiency

**DOI:** 10.3390/ani13193138

**Published:** 2023-10-08

**Authors:** Mário Quaresma, Luisa Cristina Roseiro, Tatiana Ferreira, Maria Leonor Nunes, Gonçalo Pereira

**Affiliations:** 1 CIISA—Centre for Interdisciplinary Research in Animal Health, Faculty of Veterinary Medicine, University of Lisbon, 1300-477 Lisboa, Portugal; 2 AL4AnimalS—Associate Laboratory for Animal and Veterinary Sciences, Faculty of Veterinary Medicine, University of Lisbon, 1300-477 Lisboa, Portugal; 3 INIAV—Food Technology and Safety Division, National Institute for Agricultural and Veterinary Research (INIAV, IP), Quinta do Marquês, 2780-159 Oeiras, Portugal; 4 GeoBioTec—Geobiosciences, Geoengineering e Geobiotechnologies, NOVA School of Science and Technology, Campus de Caparica, 2829-516 Caparica, Portugal; 5 CIIMAR—Interdisciplinary Centre of Marine and Environmental Research, University of Porto, Terminal de Cruzeiros do Porto de Leixões, Av. General Norton de Matos S/N, 4450-208 Matosinhos, Portugal

**Keywords:** average daily gain, carcass weight, feed conversion ratio, oat hay, rabbit production, whole carrot

## Abstract

**Simple Summary:**

Rabbit meat production presents high labor costs per rabbit, making it less competitive than other meats. Concentrate feed, the prime feeding option in rabbit meat production represents an important share of total production cost. Therefore, to reduce rabbit meat production costs and make it more competitive, it is essential to incorporate alternative feed sources with reduced costs. The study aimed to investigate the effect of supplementation with oat hay and whole carrot from self-production, presented singly or in combination on rabbit production efficiency. The results revealed that combined supplementation with oat hay and whole carrot resulted better than supplementation presented independently and reduced concentrate intake (39.2% less or minus 1107 g/animal) without affecting carcass meat weight. At the same time, supplementation with oat hay represented the lowest feeding cost of all in the analysis (less than 14% of the cost associated with feeding exclusively on concentrate feeding). Therefore, the supplementation of rabbit’s diet either with oat hay singly or in combination with whole carrot, from self-production, is an effective option to reduce rabbit’s production cost.

**Abstract:**

Is it possible to reduce feeding costs in rabbit meat production without compromising rabbit health and productive yield? The study tested four feeding strategies: Control group (CC) fed exclusively with concentrate feed; group CT supplemented with whole carrot; group OH supplemented with oat hay; and Group CO supplemented with oat hay and whole carrot. Each feeding strategy was tested in 20 rabbits, randomly allocated in five cages with four rabbits each. The average daily weight gain (ADG), feed conversion ratio (FCR), and the amount of concentrated feed consumed daily were estimated in all experimental groups. Group CC displayed the best ADG (37.1 g/rabbit/day), carrot had no significant influence on ADG (34.2 g/rabbit/day), but oat hay had a negative impact (*p* < 0.05), either used alone or in combination with carrot (33.0 and 32.6 g/rabbit/day, respectively). Supplementation with carrot, oat hay, or both increased the FCR (*p* < 0.001). Nevertheless, there were no significant differences in final live weight or carcass weight between the rabbits in the different experimental groups. In conclusion, supplementation with oat hay, carrot, or both can be a valid approach to reducing production costs by decreasing concentrate feed without affecting rabbit’s health and meat yield. The combined supplementation with oat hay and carrot proved to be the best option in reducing the amount of concentrate feed ingested by rabbits (less than 1107 g/animal), but at current market values, supplementation exclusively with oat hay was the less expensive feeding strategy (less 14% than fed exclusively with concentrate feeding).

## 1. Introduction

The domestic rabbit (*Oryctolagus cuniculus*) is considered both a pet and a production species. In 2021, nearly 572 million rabbits were slaughtered worldwide, providing more than 861 thousand tons of rabbit carcasses [1], and rabbit meat production was concentrated in Asia (66.1%), Europe (17.3%), and Africa (14.4%), accounting for 97.8% of the overall production. The remaining 2.2% of the global rabbit production was located in the Americas, mostly in Latin America [1]. According to the analysis of world meat production, rabbit is one of the least used species for this purpose, and in 2018, rabbit meat accounted for only 0.45% of total meat production worldwide [2], with an annual per capita consumption of 0.19 kg. In comparison, pork and poultry were the first and second most consumed meats globally (15.83 and 14.35 kg/capita, respectively) [3]. Nevertheless, in the European Union, rabbit meat consumption is higher, with an annual per capita consumption of 1.7 kg, but it remains one of the least consumed meats, accounting for only 1–2% of all meat consumed [4].

The domestic rabbit is classified as a non-ruminant herbivorous and hindgut fermenter species with caecotrophy [5,6]. These characteristics allow the rabbit’s diet to be predominantly composed of forages and by-products [7], and it is therefore considered a non-competitive species with human food [5]. The microbiota in the caecum is able to partially hydrolyze dietary fiber and synthesize volatile fatty acids, protein, and vitamins, which are stored in caecotrophs, also known as soft feces, which are ingested directly from the anus, allowing the rabbit to use these nutrients [8]. Caecotrophs are rich in microbial proteins, with essential amino acids, and have B-complex and K vitamins [8].

Rabbit meat production presents high labor costs per rabbit, making the cost of production higher than those of broiler and pig meat, which makes rabbit meat less competitive [9,10,11]. Therefore, it is essential to reduce the rabbit meat production cost. However, almost one-third of the production depends on feed consumption by the fattening unit, which accounts for 50–60% of all the feed consumption in the rabbit production cycle [12]. Therefore, feed cost and feed conversion rate in the rabbit fattening units are of primary importance for profit [12,13].

In ruminant production, the self-production of fodder is a primary goal to achieve total or partial feeding sustainability since it allows lower feed costs and complete control of the forage used in animal feeding. However, this objective is less common in monogastric species, such as pigs, poultry, and rabbits, as they rely on a concentrated feed that provides them with all the nutrients in the amounts required to achieve full efficiency. Nevertheless, reducing feed costs is of paramount importance in rabbit meat production. To achieve this objective, it is essential to study feed alternatives herein; the self-production of oat hay (*Avena sativa*) and whole carrot (*Daucus carota*) were regarded as reliable feeding options for rabbit production, considering the farm conditions, the need to reduce feeding costs and improve farm sustainability.

The carrot (*Daucus carota*) is a popular root vegetable grown and consumed worldwide by humans. Rich in phytonutrients with health-promoting properties, such as carotenoids (particularly the α- and β-carotene, both with pro-vitamin A effect), phenolic compounds with antioxidant potential, and dietary fiber (an essential element in rabbits diet). Beyond phytonutrients, carrot is a source of both macro- and microminerals (as calcium, phosphorus, iron, magnesium, sodium, potassium, zinc, and copper) and vitamins (B1, B2, B3, B9, and C [14,15]). Regarding carrot’s fiber composition, it is constituted predominantly cellulose (80.94% of dry weight), followed by hemicellulose (9.14% of dry weight), pectin (7.41% of dry weight), and lignin (2.48% of dry weight) [16]. Oat hay has high fiber content, which is essential for rabbits’ dental and gastrointestinal health; the incorporation of oat hay in rabbit feeding has been previously evaluated with good results once it was concluded that self-production of hay and its inclusion in rabbit feeding was an affordable alternative to optimize the rabbit production costs [17].

In line with the necessity of feeding cost reduction in rabbit production, the present study aimed to evaluate the effect of supplementing the diet with oat hay and whole carrot (from self-production), supplied alone or together, on the production efficiency of rabbits.

To the best of our knowledge, this experimental trial is original once: (1) the supplementation options are fully dependent on farm self-production; (2) it uses the whole carrot and oat hay without any technological treatment or diet adjustments; (3) this strategy was never tested. The simplicity of the experimental design allows its replication in rabbitries in other geographies.

## 2. Materials and Methods

### 2.1. Experimental Design, Animals, Housing, Feeding and Sampling

The experimental trial was executed in accordance with the good practices stipulated in national law (decree-law nº 113/2013), which transposes the Directive 2010/63/EU of the European Parliament and of the Council of 22 September 2010 [1], and it was approved by the Organ Responsible for Animal Welfare in the Veterinary Medicine Faculty of Lisbon University (ORBEA; REF003/2023).

The experimental trial was carried out in a commercial rabbitry (Agrodunas Lda, Cantanhede, Portugal) under commercial production conditions, i.e., those regularly used in the grow-out-fattening unit, namely: temperature between 16–22 °C, average relative humidity of 60%; and a 12 h photoperiod, adjusted to the light cycle (7.30–19.30 h). Rabbit handling and weighing procedures were performed by a collaborator previously informed and instructed in the handling of rabbits in accordance with animal welfare standards and with respect to European regulations for rabbit production [2]. 

The 80 rabbits used in this study were commercial hybrids of Hyla 2000 rabbits (Eurolap, Gosne, France), selected at weaning (30 days old) from 10 independent litters born on a single day with a minimum of 10 healthy offspring. From each litter, eight kits were selected for the study according to their sex (sex ratio 1:1) and live weight at weaning (excluding the lightest and heaviest rabbits in the litter). The selected littermates were randomly distributed by the four experimental groups (2 littermates/experimental group with a sex ratio of 1/1) and tattooed for identification purposes in their left ear. Therefore, each experimental group encloses two littermates from 10 independent litters; a total of 20 rabbits were allocated to 5 topped wire cages with wire-mesh floor (4 rabbits per cage with 0.5 m^2^), in accordance with European regulations for rabbit production [2]. The cages included automatic feeders and drinkers. The concentrated feed was provided ad libitum at the feeders, while supplementation with oat hay or/and whole carrot (referred to here as carrot) was provided on the cage’s floor in the corner adjacent to the feeder.

Regarding dietary management, rabbits in all four experimental groups were fed with concentrate feed, presented ad libitum, and the amount of concentrate feed ingested was measured every 2 or 3 days in all 20 cages. The control group (CC group) was fed concentrate feed exclusively; the CT group was supplemented with whole carrots (50 g of carrot/rabbit/day); the OH group was supplemented with oat hay (30 g of oat hay/rabbit/day); the CO group was supplemented with oat hay and carrot (30 g of oat hay plus 50 g of carrot/rabbit/day). Table 1 presents the proximate composition of concentrate feed, oat hay, and carrot, and the ingredients used in the concentrate feed production and their percentage in the concentrate feed are shown in Table 2.

The amount of concentrate feed daily-ingested (CFDI) by the rabbits in each cage was recorded following the equation:

CFDI = (TACF − ACFL)/(nR × nD)
TACF = total amount of concentrate feed provided to the feeder on the days of supply (Mondays, Wednesdays and Fridays);
ACFL = the amount of concentrate feed left in the feeder by the early morning of days of control (Mondays, Wednesdays and Fridays);
nR = number of rabbits inside the cage (4);
nD = number of days between weighing (2 days during the week and 3 days over the weekend)

The rabbits were individually weighted on a weekly basis; for this purpose, a transport backpack for cats with breathable mesh (Pecute, Seattle, WA, USA) was used. Regarding the weighting procedure, the carrying bag was placed on the scale set to zero, and after placing the rabbit in the box, the rabbit’s weight was recorded according to the rabbit identification number. The estimated average daily weight gain (ADG; calculated individually for each rabbit) and the feed conversion ratio (FCR; estimated per cage) were calculated according to the equations:

ADG = weight difference of a single rabbit between two consecutive weighings/number of days between weighings (7 days).

FCR = feed intake in g/body weight gain in g (estimated on a weekly basis).

To evaluate the amount of oat hay and carrot ingested by the rabbits, the experimental design considered the daily supplementation of a fixed and predefined amount of both. The oat hay and carrot leftovers from group CO cages were removed and weighed daily before adding a new dose. The carrot and oat hay leftovers in the experimental groups that received them alone (CT and OH groups) were not recorded, as they were felt through the wire mesh of the cage.

The rabbits in this study were weaned at 30 days of age and entered the experimental trial immediately, but the data collection began on day 36th. The period between days 30 and 36 was not included in the experimental trial to avoid the impact of stress associated with weaning, diet changing, and collecting kits from different litters. All rabbits were weighed on the 36th day of life, and no significant difference (*p* = 0.90) was observed between the different groups regarding weight (1112.5 ± 95.4 g; mean ± standard deviation).

The rabbits were slaughtered in the Litoral Coelho slaughterhouse (Tocha, Portugal) in full compliance with the EU legislation. The carcasses, after being fully processed, were weighed, and their weight was recorded according to the rabbit identification.

### 2.2. Statistical Analysis

The statistical analysis was accomplished using the MIXED procedure of SAS (SAS Inst., Cary, NC, USA), version 9.4. The model considered a single effect (diet). The study encloses four experimental groups (diets), each one with five cages (experimental unit). The rabbit’s cage was established as the experimental unit, and rabbits within a single cage were treated as repeated measures. Statistically significant differences were considered when *p* < 0.05. The least-square means and standard error of the mean (SEM) are presented in tables.

## 3. Results

### 3.1. Feed Consumption

Table 3 presents the estimated amount of feed consumed by rabbits throughout the growing period (between 36 and 67 days of age), the total amount of concentrate feed, the total amount of dry matter (DM), the total amount of protein, the total amount of fiber, all values expressed in g/animal. Table 3 also shows the change in the total amount of feed/nutrients ingested (expressed as a percentage of the amount ingested by rabbits in the experimental groups compared to the amount ingested by the rabbits in the control group).

Group CC was fed ad libitum with concentrate feed exclusively, and the rabbits in this group ingested an average of 2821 g/animal throughout the growing period in analysis, which was considered 100%.

The CT group was fed concentrate feed, offered ad libitum, and supplemented with carrot (1600 g carrot/rabbit or 50 g carrot/rabbit/day). This mixture led to an increase in the total amount of feed ingested (averaging 33.9%; 957 g/animal), which resulted in a reduction in the amount of concentrate feed ingested relative to the CC group (22.8%; less 643 g/animal). The oat hay supplementation (associated with concentrate feed, presented ad libitum) used in the OH group (960 g oat hay/rabbit or 30 g oat hay/rabbit/day) resulted in an increase in the total amount of feed ingested by 11.4% (323 g/animal more), but reduced the amount of concentrate feed consumed, by 22.6% (less 637 g/animal). The diet used in the CO group, concentrate feed (offered ad libitum) supplemented with oat hay and carrot (960 g oat hay plus 1600 g carrot/rabbit or 30 g oat hay plus 50 g carrot/rabbit/day), resulted in an increase (51.5%) in the amount of total feed intake over the growing period (1453 g more/animal), but reduced the amount of concentrate feed by 39.2% (less 1107 g/animal).

### 3.2. Diet Composition

Due to the variability of the water content in the feed concentrate and in the supplements (oat hay and carrot), it was important to analyze the diet ingested in terms of dry matter (DM). Table 3 presents the DM intake protein, starch, fat, and ash (expressed in g/animal) by the rabbits of the different groups throughout the 32-day period.

The rabbits in the CC group, fed exclusively with concentrated feed, took in an average of 2539 g of DM per animal, equivalent to a regular daily consumption of 79.3 g/animal over the period under study. On the other hand, in the rabbits of the CT group, there was a total reduction of 385 g of DM/animal in the period considered, which resulted in a reduction of 15.2% compared to the CC group. In contrast, in the rabbits of the OH group, there was an overall average increase of 217 g of DM/animal, which gave rise to an increase of 8.5% compared to the CC group. The rabbits in the CO group had a lower intake of DM compared to the CC group (0.5% less; 12.6 g/animal).

Regarding the contribution of different feeds to the diet total DM, the concentrate feed is the prime feed responsible for the diet DM, representing 100%, 91%, 71%, and 61% of diet DM of groups CC, CT, OH, and CO, respectively. Carrot was responsible for 9% and 8% of total DM in groups CT and CO, respectively. Meanwhile, oat hay was accountable for 29% and 31% of total DM in groups OH and CO, respectively.

Beyond the total amount of feed and DM intake, the total protein intake is also of prime importance in growing animals. Assuming that concentrate feed protein content provides 100% of the rabbit growth requirements, the introduction of carrot or oat hay reduces the diet’s total protein content to 84% and 93%, respectively, whereas the combined introduction of oat hay and carrot reduces the diet’s total protein content to 84% of the Control diet’s total protein content. Carrots contributed 8% of the diet’s total protein in groups CT and CO, while oat hay was accountable for 18% and 19% of the total protein content in groups OH and CO.

### 3.3. Productive Efficiency

The rabbit’s average weight gain (ADG), presented as weekly and overall averages, is shown in Table 4. Diet significantly influenced the overall ADG (*p* = 0.002), and the highest ADG was achieved by rabbits in the CC group (averaging 37.1 g/rabbit/day). A diet supplemented exclusively with carrots had no significant influence on the overall ADG (34.2 g/rabbit/day). However, supplementation with oat hay had a negative influence (*p* < 0.05) on global ADG, regardless of whether supplemented alone or in conjugation with carrot (averaging 32.8 g/rabbit/day). In addition to the results previously presented, it was possible to evaluate the effect of different supplementation options on rabbit growth. Supplementation with oat hay and carrot used alone or in combination contributed to an ADG drop of 2.9, 4.1, and 4.5 g/day in CT, OH, and CO, respectively (minus 8, 11.1 and 12.1% of the value that was observed in the Control group). Diet significantly influenced the global ADG, but its temporal influence was limited to the second and fifth weeks (*p* < 0.05), whereas no significant influence was observed in the first, third, and fourth weeks (*p* > 0.05).

**Figure 1:**
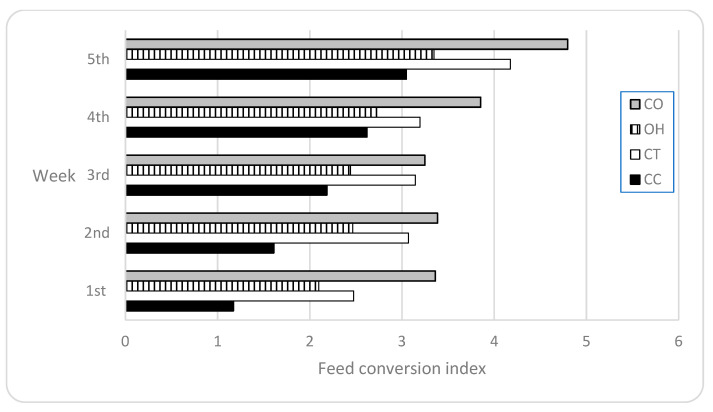
Feed conversion ratio (FCR) of rabbits in different diets throughout the 5-week period (expressed weekly as g of feed intake per g of body weight gain). Regarding experimental groups, CC stands for Control group, CT stands for carrot supplementation, OH stands for oat hay supplementation, and CO stands for carrot plus oat hay.

The influence of diet on feed conversion ratio (FCR), presented in Figure 1, shows that the Control group had the lowest FCR (best efficiency) of all experimental groups (averaging 2.13 and ranging from 1.17 in the first week to 3.05 in the last week). On the other hand, supplementation with oat hay and carrot was associated with the highest FCR (worst efficiency) throughout the experimental trial (averaging 3.73, ranging from 3.25 to 4.80). However, supplementation with oat hay or carrot resulted in the second lowest and second highest FCR of the four diets (averaged 2.60 and 3.16, respectively).

Table 5 shows the final live weight (FLW) at slaughter time, carcass weight (CW), and total weight gain (TWG) over the growing period. Supplementation of the rabbit’s diet with carrot, oat hay, and a combination of both significantly (*p* = 0.002) reduced the average TWG, a reduction of 138, 147, and 149 g, which represented the loss of 11.5%, 12.4% and 12.6% of the potential TWG. Despite the significant differences in the TWG, no significant differences were observed on both the FLW (*p* = 0.252) and CW (*p* = 0.443), which averaged 2340 and 1449 g, respectively, corresponding to a carcass yield of 61.9%.

The rabbit’s diet supplementation with whole carrot and oat hay reduced the ingestion of concentrate feed and reduced the diet cost once the whole carrot and oat hay had a market value below concentrate feed, representing just 35.4% and 25.3% of the concentrate market value, respectively. Therefore, the growth and fattening period in analysis (length of 32 days) was associated with a feeding cost of 1.12 EUR/rabbit (CC group), 1.09 EUR/rabbit (CT group), 0.96 EUR/rabbit (OH group) and 1.0 EUR/rabbit (group CO). The same is to say that the feeding cost is reduced by 2.7% with carrot supplementation, 14.0% with oat supplementation, and 10.6% with the combined supplementation with carrot and oat hay. Regarding the reduction in concentrate feed throughout the growth and fattening period in analysis, supplementation with oat hay and carrot presented singly were associated with a reduction of 637 and 643 g/rabbit, respectively, while the conjugation of carrot and oat hay was associated with a concentrate reduction of 1107 g/rabbit. Despite the absence of significant differences between experimental groups in carcass weight, supplementation with oat hay resulted in the smallest numerical difference with CC (20 g/carcass).

## 4. Discussion

The results obtained show that the reduction of the cost of rabbit meat production via the reduction of the amount of concentrated feed in the rabbit growth and fattening unit was achieved. When considering the reduction in concentrate feed ingestion, the best results were obtained with the combined supplementation of oat hay and carrot, which allowed the greatest reduction in concentrate feed (less 1107 g/rabbit), a reduction of 39.2% of the concentrate feed required per growing rabbit and a greater saving (1.73 times higher) than obtained by supplementing oat hay or carrot alone. However, supplementation with whole carrots and oat hay is also associated with a cost, even when it is from self-production. Herein, we decided to use the market value of both raw materials instead of the self-production cost to avoid the self-production cost variability between farms. Consequently, the introduction of the supplementation cost shows that, at current market values, supplementation with oat hay was the best economic option since it reduced the feeding cost of the period in analysis by 14.0%, while the combined supplementation of whole carrot and oat hay reduced the feeding cost by 10.6%.

It is possible to say that the best strategy to reduce concentrate feed ingestion is the combined supplementation of whole carrot and oat hay (saves 1107 g/rabbit), but the best option to reduce the feeding cost in the growth and fattening period is the supplementation with oat hay (saves 637 g/rabbit and reduces the feeding cost in 14%).

This experimental trial ended with a mortality rate of 0% in all experimental groups, an important result considering the rabbit’s gastrointestinal susceptibility [18]. Although the main objective was achieved, the imbalance between the diet composition and the nutritional needs of rabbits is considered a major constraint to rabbit growth, which is reflected in lower ADG and higher FCR.

Total protein content is fundamental for animal growth, and the combined supplementation with oat hay and carrot contributed to a considerable reduction in the total amount of protein intake (16.1% less than that observed in Group CC). In comparison, supplementation with oat hay resulted in the lowest fall in protein intake (6.3% less than in Group CC). The fall in total protein intake may negatively influence the amount of digestible protein and the bioavailability of essential amino acids, with negative consequences for the growth of rabbits.

In the present study, the combined supplementation with oat hay and carrot was associated with a significant reduction in ADG compared to Group CC (less 4.5 g/rabbit/day) and an increase in FCR (75% more). On the other hand, supplementation with oat hay resulted in a minor reduction in the ADG (less than 4.1 g/rabbit/day) and a minor increase in the FCR (35% more). Such differences may be the result of the diet’s lower protein content, a consequence of whole carrot and oat hay lower protein content than concentrated feed. Our results do not corroborate the compensatory mechanism of nitrogen transfer in the rabbit’s digestive system when fed low protein content diets previously presented [19]. On the other hand, reducing the dietary protein content decreases ileal protein flow and reduces pathogens proliferation and mortality during the fattening period [20], which is in agreement with the absence of deaths among the rabbits used in the trial.

Beyond lower protein content, the combined supplementation with oat hay and carrot contributed to a considerable increase in total fiber, fat, and starch intakes (73.5%, 62%, and 48% more than observed in Group CC, respectively), whereas supplementation with oat hay contributed to an increase in the total fiber and total fat ingested (an increase of 47.9 and 8.8%, respectively) but was associated with a decrease in the amount of starch and ash ingested −1.3 and −5.4%, respectively.

Fiber is one of the main components of the concentrated feed used in the intensive production of rabbit meat, as it is essential to maintain gut health [5]. The importance of fiber in the rabbit’s diet relies on several key functions, including nutrient supply, stimulation of gut motility (insoluble fiber only), maintenance of intestinal mucosa integrity, caecal fermentation, and regulation of microbiota [5,21]. Despite fiber’s key role in a rabbit’s digestive system functioning, a fiber content above nutritional needs leads to energy dilution of the diet, conditioning a higher food intake with the consequent increase in the FCR. However, such compensatory feeding behavior cannot handle very high fiber contents (>25% ADF) [22].

As in other herbivorous species, fiber digestion is the responsibility of microbial fermentation, which in the rabbit occurs predominantly in the caecum. However, the caecal contents are emptied daily to produce caecotrophs, which limits caecal retention/fermentation to a short period (nearly 10 h). Consequently, the cellulolytic activity of the caecum microbiota is quite low but is able to degrade significant amounts of more soluble non-starch polysaccharides [21,22,23]. The post-weaning period in rabbits, as in other monogastric herbivores, requires caution due to the incomplete development of the enzymatic system and the rapid transit flow. Therefore, dietary references advocate a low dietary amount of starch avoid digestive disorders since undigested starch in the small intestine is fermented by the microbiota in the caeco-colic segment, with possible negative results on digestive function [24].

Nevertheless, rabbits appear to be highly efficient in starch digestion, even in those starch sources known to be poorly digestible in monogastric species; such efficiency is corroborated using the high ileal digestibility of starch, averaging 0.94 [25,26]. The amount of fermented starch in the caeco-colic segment of 30–60 days old rabbits varies between 3 and 11% of the total starch intake [26]. Thus, in rabbits, starch may represent an important source of energy, and diets with higher starch content resulted in higher body weight gain and better feed conversion without significant negative consequences on digestive physiology [25]. In comparison with Group CC, the introduction of whole carrot contributed to an increase in the starch content in the diet when presented singly or in conjugation with oat hay (more than 43.2% and 48%, respectively), but such increase in diet’s starch content had no apparent negative influence on rabbit growth.

Regarding the fat component of the diet in growing rabbits, they require small amounts of essential fatty acids, while nonessential fatty acids are a source of energy, stimulate the absorption of fat-soluble biomolecules, and provide desirable textural and flavor characteristics to the feed [27,28,29]. Increasing the diet’s total fat content has been associated with advantages and disadvantages. Higher fat content improves FCR and overall protein retention efficiency [28], improves body condition, stimulates the immune system, and improves health status [30]. On the other hand, higher fat contents have also been associated with lower digestive efficiency and reduced microflora activity in the caecum [31].

Regarding the dietary supplementation with oat hay on rabbit’s growth and productive efficiency, when applied singly, there is an FCR rise of 35.1% and an ADG drop of 11.1%, but when applied in conjugation with whole carrot, there is an FCR rise of 75.3% and an ADG drop of 12.1%. Therefore, it is possible to say that supplementation with oat hay contributes to worsening productive efficiency, and the combined supplementation with oat hay and carrots further worsens the productive efficiency. Nevertheless, no significant influence was observed on the rabbit’s final live weight and carcass weight, which is what matters in rabbit meat production. Despite the absence of significant differences between experimental groups on carcass weight, supplementation with oat hay presented singly resulted better than when combined with whole carrot, once the carcass weight drop was of just 20 g/carcass comparatively to 69 g/carcass.

## 5. Conclusions

Supplementing fattening rabbits with oat hay, carrot, or a combination of both resulted in a reduction in concentrate intake of 22.6%, 22.8%, and 39.2%, respectively. Although carrot supplementation did not affect ADG, oat hay supplementation had a negative impact, whether used alone or in combination with carrot. Furthermore, supplementation with carrot, oat hay, or both increased the FCR, indicating a decrease in production efficiency. Despite the negative impact on ADG and FCR, there were no significant differences in final live weight or carcass weight among the groups in comparison. Therefore, supplementing with oat hay, carrot, or both may be a valid approach to reduce production costs by decreasing concentrate feed without affecting rabbit meat yield.

The combined supplementation with oat hay and whole carrot was the best option to reduce concentrate feeding ingestion, but presently, oat hay presented singly was the best way to reduce the rabbit’s diet cost.

## Figures and Tables

**Table 1:** Proximate composition of the concentrate feeding, carrot, and oat hay used in the experimental trial and their actual market value (expressed as EUR/ton).

	Concentrate	Carrot	Oat Hay
Raw material cost (EUR/ton)	369	140	100
DM (% of TW)	90.0%	12.1%	82.3%
Crude protein (% of DM)	14.0%	1.7%	6.7%
Crude fiber (% of DM)	15.2%	11.3%	31.5%
ADF (% of DM)	20.0%	28.5%	32.9%
NDF (% of DM)	32.8%	38.4%	68.0%
Total fat (% of DM)	2.6%	3.2%	2.4%
Starch (% of DM)	10.4%	12.1%	6.5%
Ashes (% of DM)	9.7%	6.1%	4.9%

% of TW—expressed as % of total weight; % of DM—expressed as % of total dry matter weight.

**Table 2:** Ingredients and chemical composition of the concentrate feeding used in this experiment (presented as a percentage of total feed).

Ingredient	%
Sunflower meal < 30% CP	26.00
Alfalfa meal	20.82
Wheat bran	15.63
Barley	11.78
Dried citrus pulp	6.00
Sugar beet pulp	5.42
Palm kern meal	4.50
Sugarcane molasses	3.00
Sepiolite	2.00
Lime fine	1.32
Sea salt premix	1.17
Carrob fruit	0.83
Lignosulfonate	0.63
Vitamine and minerals premix	0.40
Lard	0.25
L-lysine (99%)	0.15
L-threonine (99%)	0.06
DL-methionine (99%)	0.02
Grass aroma	0.01

**Table 3:** Total feed, total concentrate feeding, and total DM (total protein, fiber, fat, starch, and ash) were ingested by rabbits throughout the growing period (expressed as g/rabbit or as % of the Control group).

	Group			
	CC	CT	OH	CO
Feed provided, g/rabbit	2821	3778	3144	4274
Total feed, % ^1^	100	133.9	111.4	151.5
Total concentrate ingested, g/rabbit	2821	2178	2184	1714
Total concentrate, % ^1^	100	77.2	77.4	60.8
Total DM ingested, g/rabbit	2538.9	2153.8	2755.7	2526.3
Total DM, % ^1^	100	84.8	108.5	99.5
Total protein ingested, g/rabbit	394.9	332.1	370.1	331.5
Total protein, % ^1^	100	84.1	93.7	83.9
Total fiber ingested, g/rabbit	428.8	511.9	634.4	743.7
Total fiber, % ^1^	100	119.4	147.9	173.5
Total fat ingested, g/rabbit	73.3	107.8	79.8	118.8
Total fat, % ^1^	100	147.0	108.8	162.0
Starch ingested, g/rabbit	293.4	420.1	289.5	434.3
Starch, % ^1^	100	143.2	98.7	148.0
Ash ingested	273.6	308.9	258.9	310.9
Ash, % ^1^	100	112.9	94.6	113.6

CC stands for control group, CT stands for carrot supplementation, OH stands for oat hay supplementation, and CO stands for carrot plus oat hay. ^1^ The total amount provided is a percentage of the total amount provided to CC group.

**Table 4:** Daily weight gain throughout the experimental trial (expressed as g/rabbit/day) by week and throughout the experimental period.

Average Daily Weight Gain	Group					Statistics	
	CC	CT	OH	CO		SEM	*p*
1st week	41.7	39.7	37.7	37.5		1.69	0.267
2nd week	32.9 ^a^	28.5 ^a,b^	29.9 ^a^	26.9 ^b^		1.33	0.014
3rd week	42.7	39.8	38.0	41.2		2.05	0.435
4th week	34.5	34.2	32.3	32.3		1.57	0.639
5th week	33.5 ^a^	28.5 ^a,b^	27.3 ^b^	25.4 ^b^		1.53	0.003
Total	37.1 ^a^	34.2 ^a,b^	33.0 ^b^	32.6 ^b^		0.84	0.002

Regarding experimental groups, CC stands for Control group, CT stands for carrot supplementation, OH stands for oat hay supplementation, and CO stands for carrot plus oat hay. Different superscripts differ significantly (*p* < 0.05).

**Table 5:** Final live weight at slaughter time, total weight gain throughout the growing period, and carcass weight.

	Group					Statistics	
	CC	CT	OH	CO		SEM	*p*
Total weight gain, g	1336 ^a^	1198 ^b^	1189 ^b^	1187 ^b^		31.34	0.002
Final live weight, g	2357	2335	2392	2276		41.58	0.252
Carcass weight, g	1490	1432	1470	1421		33.99	0.443

Regarding experimental groups, CC stands for Control group, CT stands for carrot supplementation, OH stands for oat hay supplementation, and CO stands for carrot plus oat hay. Different superscripts differ significantly (*p* < 0.05).

## Data Availability

The data that support the findings of this study are available from the corresponding author upon rational request.
